# Emergence of High-Level Cefiderocol Resistance in Carbapenem-Resistant Klebsiella pneumoniae from Bloodstream Infections in Patients with Hematologic Malignancies in China

**DOI:** 10.1128/spectrum.00084-22

**Published:** 2022-03-24

**Authors:** Peng Lan, Ye Lu, Zhongju Chen, Xueqing Wu, Xiaoting Hua, Yan Jiang, Jiancang Zhou, Yunsong Yu

**Affiliations:** a Department of Critical Care Medicine, Sir Run Run Shaw Hospital, Zhejiang University School of Medicine, Hangzhou, China; b Key Laboratory of Microbial Technology and Bioinformatics of Zhejiang Province, Hangzhou, China; c Regional Medical Center for National Institute of Respiratory Diseases, Sir Run Run Shaw Hospital, Zhejiang University of Medicine, Hangzhou, China; d Department of Laboratory Medicine, Tongji Hospital, Tongji Medical College, Huazhong University of Science and Technology, Wuhan, China; e Department of Infectious Diseases, Sir Run Run Shaw Hospital, Zhejiang University School of Medicine, Hangzhou, China; University of Guelph

**Keywords:** bloodstream infections, cefiderocol, CRKP, NDM, CirA

## Abstract

Cefiderocol is a novel siderophore cephalosporin exhibiting potent antimicrobial activities. Although cefiderocol has not been approved in China, resistance is emerging. A multicenter study was performed to evaluate the cefiderocol resistance in carbapenem-resistant Klebsiella pneumoniae (CRKP) strains from bloodstream infections in patients with hematologic malignancies in China. Clinical data analysis and whole-genome sequencing were conducted for collected cefiderocol-resistant CRKP strains. CRISPR-Cas9 system was employed to construct site-specific mutagenesis for gene *cirA*. Plasmid curing and cloning were performed to assess the effect of β-lactamases on cefiderocol resistance. Total 86 CRKP strains were collected. The MICs of cefiderocol ranged from 0.06 to >256 mg/L. Among four cefiderocol-nonsusceptible strains (4/86, 4.7%), two cefiderocol-resistant strains AR8538 (MIC = 32 mg/L) and AR8416 (MIC > 256 mg/L) were isolated from two patients with acute lymphocytic leukemia (frequency of resistance, 2/86, 2.3%). Metallo- and serine-β-lactamase inhibitors addition would decrease the MIC of cefiderocol from 32 to 1 mg/L in AR8538, which harbors *bla*_SHV-12_, *bla*_DHA-1_, and two copies of *bla*_NDM-1_ in different plasmids. Avibactam did not impact cefiderocol susceptibility of AR8416, which produces NDM-5. However, we found a deficient CirA in AR8416. Using the same K serotype strain D3, we proved CirA deficiency or carrying NDM individually reduced cefiderocol susceptibility, but their simultaneously existence rendered a high-level cefiderocol resistance. In summary, the resistance of CRKP against cefiderocol is mediated by multiple factors, including the deficiency of CirA, metallo- or serine-β-lactamases, while a high-level cefiderocol resistance could be rendered by the combined effect of NDM expression and CirA deficiency.

**IMPORTANCE** Cefiderocol-resistant CRKP strains are emerging in bloodstream infections in Chinese patients with hematologic malignancies, although cefiderocol has not been approved for clinical use in China. Our study proved that the resistance of CRKP against cefiderocol is mediated by multiple factors, including the deficiency of CirA, metallo- or serine-β-lactamases, while a high-level cefiderocol resistance could be rendered by the combined effect of NDM expression and CirA deficiency. As NDM production is one of the most critical mechanisms resulting in carbapenem resistance, it would pose great challenges on the clinical efficacy of cefiderocol in future.

## INTRODUCTION

Patients with hematological malignancies have an increased risk for bloodstream infections (BSI), which are among the most common and severe infectious complication, with a prevalence of 11% to 38% and associated with a 40% rise in crude mortality (1). Particularly, those with Gram-negative pathogens, especially carbapenem-resistant K. pneumoniae (CRKP) has become one of the major emerging causes of fatal infections in patients with hematological malignancies (1, 2). Due to the increasing prevalence of multidrug-resistance (MDR), the treatment of infections caused by CRKP rely on tigecycline, polymyxins or new β-lactam/β-lactamase inhibitor combinations (such as ceftazidime-avibactam, CAZ/AVI) (3, 4). Unfortunately, the resistance against these antimicrobials, the last-resort therapy, is already increasingly emerging in CRKP (5). Moreover, the activity of CAZ/AVI against class B β-lactamase (eg, NDM) is limited (6). An effective antimicrobial alternative is therefore urgent.

Cefiderocol is a novel cephalosporin antibiotic showing a potent and broad-spectrum activity against Gram-negative MDR pathogens. Cefiderocol contains a catechol moiety, which mimics catecholate-type siderophores (e.g, enterobactin) to chelate ferric iron (7). To acquire iron, bacterial cells would actively transport cefiderocol-Fe^3+^ complexes across outer membrane into the periplasmic space via iron transport systems. This “trojan horse strategy” renders increased periplasmic concentrations of cefiderocol, thus exhibiting potent activity against various carbapenemase-producing Gram-negative bacilli (8, 9). In spite of the short-term usage, strains with reduced susceptibility to cefiderocol have been reported. For example, amino acid deletions in the R2 loop of AmpC β-lactamase conferred increased hydrolysis of cefiderocol, resulting in decreased susceptibility to cefiderocol but not yet reaching to resistant level (10, 11). NDM and PER were also reported contribute marginally to the decrease of the susceptibility to cefiderocol but none of them could lead to cefiderocol resistance (12). It indicated that β-lactamase production was not fully responsible for cefiderocol resistance. Meanwhile, mutations of *cirA* gene (encoding catecholate siderophore receptor) were observed in NDM-producing cefiderocol-resistant Enterobacter cloacae strains (13), but no change of cefiderocol susceptibility was observed in *cirA* knocked out E. coli (14). Therefore, the role of CirA in mediating cefiderocol resistance remains ambivalent.

The Food and Drug Administration (FDA) of USA and European Medicines Agency (EMA) have recently approved the usage of cefiderocol and it will soon be approved in China. Therefore, we collected CRKP strains isolated from bloodstream infections in patients with hematologic malignancies from 15 centers in China, to evaluate their cefiderocol susceptibility and further study the mechanisms of cefiderocol resistance, with the aim of providing guidelines for clinical practice of cefiderocol.

## RESULTS

### Cefiderocol susceptibility.

**FIG 1:**
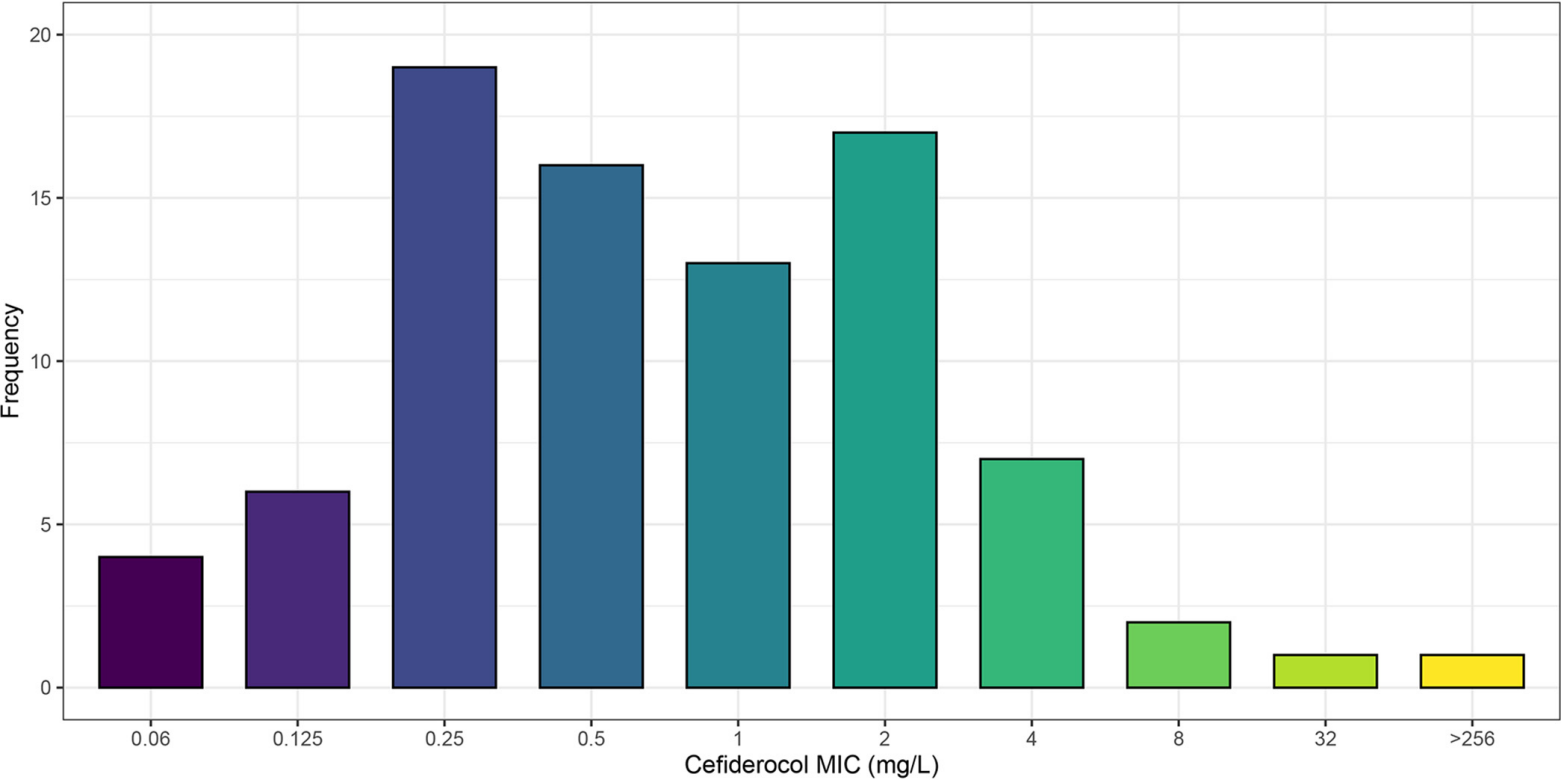
Susceptibility profile of 86 CRKP strains from bloodstream infections in hematological patients in China.

We collected a total of 86 nonrepetitive CRKP strains from blood of hematological patients, covering 15 tertiary hospitals from January 2018 to December 2020 (Fig. S1). The MICs for cefiderocol ranged from 0.06 mg/L to greater than 256 mg/L (Fig. 1 and Table S2). The MIC_50_ and MIC_90_ of cefiderocol against these strains was 0.5 mg/L and 4 mg/L, respectively (Fig. 1). Four nonsusceptible strains were identified, including two cefiderocol-resistant K. pneumoniae strains AR8538 (MIC = 32 mg/L) and AR8416 (MIC > 256 mg/L).

### Effect of β-lactamases on cefiderocol resistance.

To evaluate the effect of β-lactamase on cefiderocol resistance, cefiderocol MICs were determined in the presence or absence of β-lactamase inhibitors. Cefiderocol-nonsusceptible CRKP strains (MIC ≥ 8 mg/L) were included as shown in Table 1. All the 4 strains produced NDM-1 or NDM-5. Against 3 of 4 metallo-β-lactamase producers, decreasing 4–8-folds in MICs was observed after adding AVI except for AR8416, which still remained highly resistant in the presence of AVI. By adding DPA, a more than 8-fold decrease of cefiderocol MICs was observed and the MIC against AR8416 decreased from ≥ 256 mg/L to 1 mg/L. When adding both AVI and DPA, the MICs even decreased 16–128-folds. Combined, these results suggested both metallo- and serine-β-lactamases contributed to reduced cefiderocol susceptibility.

**TABLE 1:** *In vitro* activity of cefiderocol with or without β-lactamase inhibitors^*a*^

Strains	β-lactamase	Cefiderocol MIC (mg/liter)			
		-^*b*^	AVI	DPA	AVI+DPA
AR8416	NDM-5	>256	>256	1	1
AR8538	NDM-1 × 2, DHA-1, SHV-12	32	8	2	1
AR8334	NDM-5, SHV-208	8	2	1	0.5
AR8335	NDM-1, SHV-12, IMP-4	8	1	1	0.06

a AVI, avibactam; DPA, dipicolinic acid; MIC, minimum inhibitory concentration.

b -, No β-lactamase inhibitor was added.

### Clinical and genomic characteristics of AR8416 and AR8538.

K. pneumoniae AR8538 and AR8416 were isolated from two young males (both 20’s) who were diagnosed with acute lymphocytic leukemia in different hospitals. AR8538 was isolated after 1-day meropenem treatment. The patient infected by AR8416 received courses of tigecycline, polymyxin B, meropenem and cefperazone-sulbactam during the hospitalization. A diagnostic bloodstream culture grew AR8416 with cefiderocol MIC of >256 mg/L on day 3. Both patients experienced treatment failure eventually (Fig. S2).

**FIG 2:**
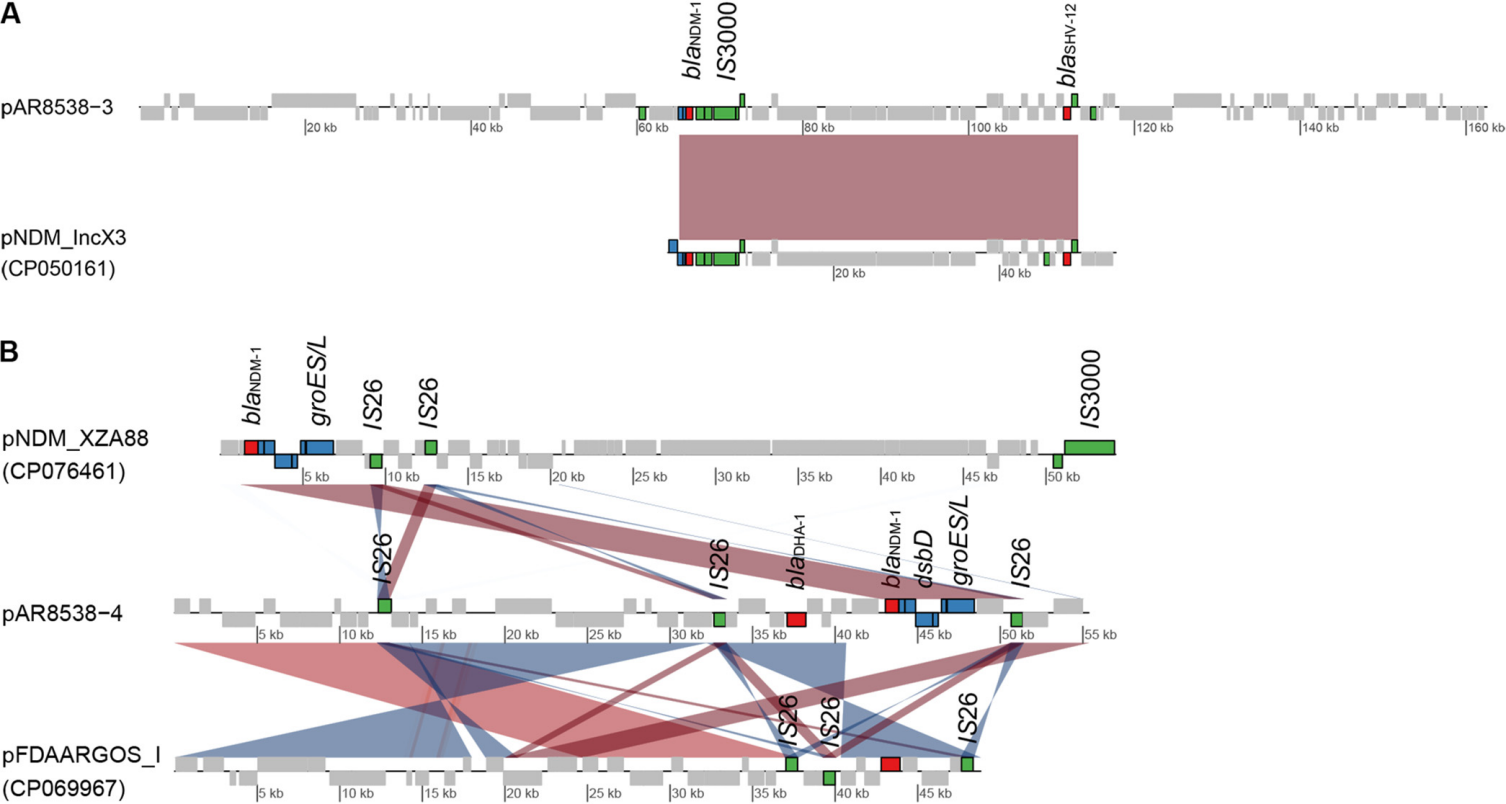
Synteny plot of plasmid pAR8538_3 and pAR8538_4. Direct comparisons are colored with red hues while reverse comparisons are colored with blue hues. Boxes on top depict CDS on the forward strand, and those at the bottom depict CDS on the reverse strand. Genes encoding β-lactamases were indicated with red, insert sequences with green and genetic context of *bla*_NDM_ with blue.

According to whole-genome sequencing data, strain AR8538 belonged to a novel sequence type, ST5820, which was a single-locus variant of ST3520 and was identified as *K. quasipneumoniae* by Kleborate. AR8538 harbored 4 plasmids with IncX3-type plasmid pAR8538_3 carrying *bla*_NDM-1_ and *bla*_SHV-12_ and IncFIA (HI1)-type plasmid pAR8538_4 carrying *bla*_NDM-1_ and *bla*_DHA-1_ (Fig. 2A and B). pAR8538_3 had 32% query coverage and 99.99% nucleotide identity with pNDM_IncX3 (CP050161), which also carried *bla*_NDM-1_ and *bla*_SHV-12_ (Fig. 2A). Another plasmid pAR8538_4 showed 82% coverage and 100% identity with pFDAARGOS_I (CP069967), which lack genetic contexts of *bla*_NDM-1_, while pNDM-XZA88 (CP076461) aligned well to genetic contexts of *bla*_NDM-1_ in pAR8538_4 (Fig. 2B). The *cirA* gene was intact in AR8538.

**FIG 3:**
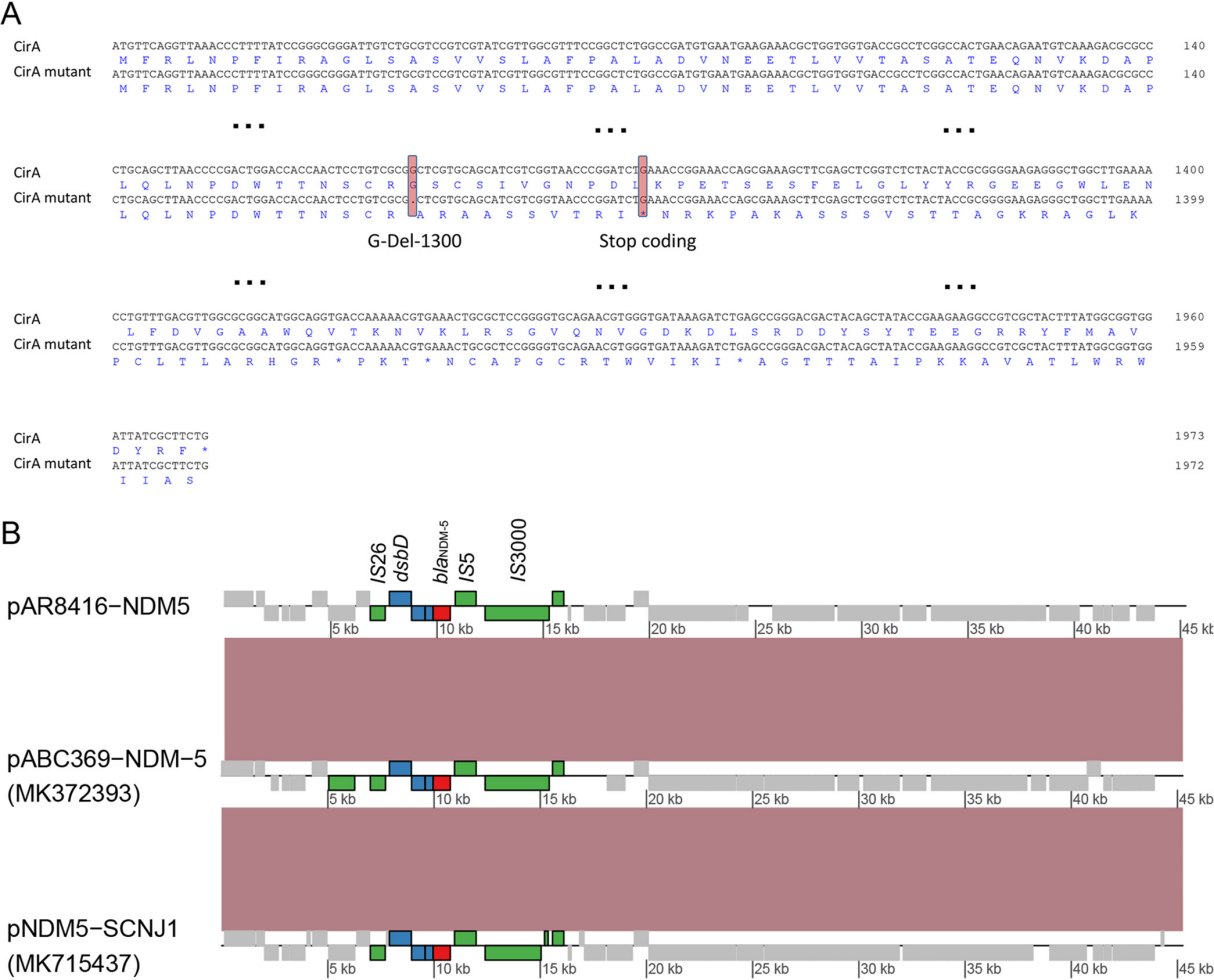
CirA deficiency and *bla*_NDM-5_-bearing plasmid in strain AR8416. (A) Alignment of intact *cirA* sequence and mutant *cirA* sequence in AR8416. The base deletion and resulting early stop codon are indicated with pink hues. (B) Synteny plot of plasmid pAR8416-NDM5 with plasmids pABC369-NDM-5 (MK372393) and pNDM5-SCNJ1 (MK715437).

Confirmed by whole-genome sequencing, AR8416 belonged to ST 5214 and K locus (KL) 1. Colicin I receptor (CirA) of K. pneumoniae consists of a transmembrane β-barrel with a luminal ‘plug’ domain positioned inside (15). Chromosomal *cirA* gene of AR8416 had a deletion of guanine base at position 1300 (Fig. 3A), rendered an early stop codon at position 444 of CirA, leading to the deficiency of β-barrel structure of CirA (Fig. S3). Among the 86 CRKP strains, the *cirA* mutation was only detected in AR8416. The strain harbored plasmid-mediated *bla*_NDM-5_, *qnrS1*, *sul2*, *floR*, *tet*(A) and chromosomal *bla*_SHV-1_. AR8416 carried 4 plasmids, with *bla*_NDM-5_ carried by a 45-kb IncX3-type plasmid, designated pAR8416-NDM5 (Fig. 3B). Insertion sequence (IS) ISS*wi1*, IS*300* and IS*5* were upstream of *bla*_NDM-5_, while *ble*_MBL_ (mediating bleomycin resistance), *trpF* (encoding a phosphoribosylanthranilate isomerase), *dsbD* (encoding a twin-arginine translocation pathway signal sequence domain protein) were downstream, which were common genetic contexts of *bla*_NDM_ (16). A copy of IS*26* was also involved in downstream *bla*_NDM-5_. BLASTN analysis revealed that pAR8416-NDM5 displayed 100% query coverage and 100% nucleotide identity with plasmid pABC369-NDM-5 (MK372393) carried by a K. pneumoniae strain in the United Arab Emirates, and plasmid pNDM5-SCNJ1 (MK715437) carried by a K. pneumoniae strain isolated in China.

### pAR8416-NDM5 is not the only factor resulting in high-level cefiderocol resistance in AR8416.

Knocking out of *bla*_NDM-5_ failed in AR8416. To explore the influence of pAR8416-NDM5 on the high-level resistance of AR8416, we obtained isolate AR8416-1 with only plasmid pAR8416-NDM5 cured. As expected, curing of plasmid pAR8416-NDM5 resulted in a sharp decrease in cefiderocol resistance (MIC, from >256 to 0.25 mg/L) (Table 2). The resistance was then reverted with the complement of pAR8416-NDM5. It was indicative that pAR8416-NDM5 might be a decisive factor for cefiderocol resistance in AR8416. However, when pAR8416-NDM5 was introduced into D3 (a ST23 and K1 serotype clinical K. pneumoniae strain isolated from liver abscess, GenBank accession number: ERS3900049) and E. coli DH5α, the cefiderocol MIC increased 8-fold (MIC, from 0.25 to 4 mg/L) and 16-fold (MIC, from 0.125 to 2 mg/L), respectively, yet still remaining susceptible. Hence, pAR8416-NDM5 reduces cefiderocol susceptibility but was not the determining factor resulting in cefiderocol resistance.

**TABLE 2:** Effect of plasmid pAR8416-NDM5 on cefiderocol susceptibility in K. pneumoniae AR8416, D3 and E. coli DH5α

Strain	Description	MIC (mg/L)^*a*^
AR8416	Wild type	>256
AR8416-1	pAR8416-NDM5 cure strain of AR8416	0.25
AR8416-2	AR8416-1 complemented with pAR8416-NDM5	>256
D3	Wild type	0.5
D3+pAR8416-NDM5	D3 transformed with pAR8416-NDM5	4
DH5α	Wild type	0.125
DH5α+pAR8416-NDM5	DH5α transformed with pAR8416-NDM5	2

a MIC, minimum inhibitory concentration.

### NDM expression and CirA deficiency jointly confer cefiderocol resistance.

While CirA is involved in the permeation of cefiderocol into bacterial cells (14), we speculated that the deficiency of CirA contributed to cefiderocol resistance in AR8416, in combination with pAR8416-NDM5. We then constructed a *cirA* mutation in D3, which was the same as that in AR8416. The CirA deficiency rendered the cefiderocol MIC against D3 increased 2-fold (MIC from 0.5 to 1 mg/L) (Table 3). While pAR8416-NDM5 was transformed into D3-*cirA*-mutant, cefiderocol MIC reached greater than 256 mg/L, suggesting that the presence of pAR8416-NDM5 and the deficiency of CirA jointly confered high-level cefiderocol resistance of AR841, but an individual factor could not have this effect. To determine whether *bla*_NDM-5_ or other genetic contexts in pAR8416-NDM5 were responsible for cefiderocol resistance, the *bla*_NDM-5_ gene derived from pAR8416-NDM5 was cloned in pCR2.1 vector. As shown in Table 3, both AR8416-1 and D3 strains with *cirA* mutation became highly resistant to cefiderocol after acquiring pCR2.1-NDM5, while wild-type D3 and DH5α still remained susceptible with pCR2.1-NDM5 transformed, though the MICs enhanced marginally. However, other types of carbapenemases, including KPC-2, IMP-4, OXA-48 and OXA-232 did not show the similar synergistic effect with the deficiency of CirA (Table 4).

**TABLE 3:** Combined effect of deficiency of CirA and NDM-5 on cefiderocol resistance

Strain	Description	MIC (mg/L)^*a*^
D3	Wild type	0.5
D3-*cirA*-mutant	D3 with *cirA* mutation	1
D3-*cirA*-mutant+pAR8416-NDM5	D3-*cirA*-mutant transformed with pAR8416-NDM5	>256
D3+pCR2.1-NDM5	D3 transformed with pCR2.1-NDM5	4
D3-*cirA*-mutant+pCR2.1-NDM5	D3-*cirA*-mutant transformed with pCR2.1-NDM5	>256
AR8416-1+pCR2.1-NDM5	AR8416-1 transformed with pCR2.1-NDM5	>256
D3+pCR2.1 vector	D3 transformed with pCR2.1 vector	0.5
AR8416-1+pCR2.1 vector	AR8416-1 transformed with pCR2.1 vector	0.25
DH5α + pCR2.1 vector	DH5α transformed with pCR2.1 vector	0.125
DH5α + pCR2.1-NDM5	DH5α transformed with pCR2.1-NDM5	2

a MIC, minimum inhibitory concentration.

**TABLE 4:** Combined effect of deficiency of CirA and other types of carbapenemases on cefiderocol resistance

Strain	Description	MIC (mg/L)^*a*^
D3+pCR2.1_NDM1	D3 transformed with pCR2.1_NDM1	4
D3+pCR2.1_KPC2	D3 transformed with pCR2.1_KPC2	2
D3+pCR2.1_IMP4	D3 transformed with pCR2.1_IMP4	0.5
D3+pCR2.1_OXA48	D3 transformed with pCR2.1_OXA48	0.5
D3+pCR2.1_OXA232	D3 transformed with pCR2.1_OXA232	0.5
D3-*cirA*-mutant+pCR2.1_NDM1	D3-*cirA*-mutant transformed with pCR2.1_NDM1	>256
D3-*cirA*-mutant+pCR2.1_KPC2	D3-*cirA*-mutant transformed with pCR2.1_KPC2	4
D3-cirA-mutant+pCR2.1_IMP4	D3-*cirA*-mutant transformed with pCR2.1_IMP4	1
D3-cirA-mutant+pCR2.1_OXA48	D3-*cirA*-mutant transformed with pCR2.1_OXA48	1
D3-cirA-mutant+pCR2.1_OXA232	D3-*cirA*-mutant transformed with pCR2.1_OXA232	1
AR8416-1+pCR2.1_NDM1	AR8416-1 transformed with pCR2.1_NDM1	>256
AR8416-1+pCR2.1_KPC2	AR8416-1 transformed with pCR2.1_KPC2	1
AR8416-1+pCR2.1_IMP4	AR8416-1 transformed with pCR2.1_IMP4	0.25
AR8416-1+pCR2.1_OXA48	AR8416-1 transformed with pCR2.1_OXA48	0.5
AR8416-1+pCR2.1_OXA232	AR8416-1 transformed with pCR2.1_OXA232	0.25

a MIC, minimum inhibitory concentration.

## DISCUSSION

Cefiderocol is a promising novel cephalosporin antibiotic showing expanded activity against Gram-negative bacteria producing all four Ambler classes of β-lactamases, including extended spectrum β-lactamases (ESBL) and carbapenemases. In meropenem-nonsusceptible strains in SIDERO-WT study, more than 99% *Enterobacterales* and Pseudomonas aeruginosa were susceptible to cefiderocol (17). In SIDERO-CR study, the susceptibility rate for meropenem-nonsusceptible is 97% (18). Cefiderocol-resistant strains are uncommon, and the underlying mechanisms have been little elucidated. In this study, we reported for the first time that NDM and the deficiency of catecholate siderophore receptor CirA individually contributed to reduced cefiderocol susceptibility, while combining these two factors resulted in a high-level cefiderocol resistance in K. pneumoniae.

Until now, a variety of factors have been demonstrated to be associated with reduced cefiderocol susceptibility but limited data presents cefiderocol resistance mechanism. Among which, β-lactamase production is considered to be an important reason for cefiderocol resistance. For NDM-producing K. pneumoniae strains, individual serine-β-lactamase inhibitor (AVI) or metallo-β-lactamase inhibitor (DPA) exhibits little effect on cefiderocol susceptibility (12). However, when both AVI and DPA were added for one strain, the MIC decreased 16-fold (from 4 to 0.25 μg/mL) (12). These results suggest that both metallo- and serine-β-lactamases contribute to cefiderocol resistance. Even so, different types of β-lactamases present versatile hydrolysis ability against cefiderocol. In present study, IMP4, OXA-48 and OXA-232 had no effect on cefiderocol susceptibility while cefiderocol MIC against D3 increased from 0.5 to 2 mg/L after acquiring KPC-2. It suggested KPC-2 might possess a higher hydrolysis ability against cefiderocol, though KPC-2 is not sufficient to result in cefiderocol resistance, even plus CirA deficiency. In contrast, NDM-5, as well as NDM-1, is the most effective β-lactamase contributing to cefiderocol resistance in our tested lactamases. Our results support previous findings that NDM confers greater MIC_50_ values (2 μg/mL) than KPC, GES, IMP, VIM and OXA (0.12 - 1 μg/mL) (8) and NDM-producers showed an MIC distribution of cefiderocol with a higher concentration range compared with other carbapenemase producers (19). Interestingly, a recent study demonstrated that NDM but not KPC-2 and OXA-48 facilitate the acquisition of CirA mutation (20). Further kinetic parameters are required to explain the superiority of NDM. Worryingly, recent measurements from Poirel L et al. showed that the specific activities against cefiderocol of β-lactamase PER-1 is greater than NDM-1 (2.14 *versus* 0.28 μmol/min·mg) (21). It is noteworthy that PER might cause threat for future cefiderocol resistance.

*bla*_NDM_ in the two cefiderocol-resistant strains were all located on plasmids. Different from pAR8416-NDM5, the *bla*_NDM_-bearing plasmids in AR8538 were less common. Plasmid pAR8538_3 is a large recombinant while the *bla*_NDM-1_ of pAR8538_4 might be acquired via IS26-mediated translocatable unit mobilization. Interestingly, AR8538 harbored two copies of *bla*_NDM-1_ located on different plasmids, which was seldom reported. In addition to NDM-1, DHA-1 and SHV-12 also contributed to cefiderocol resistance of AR8538. It suggested that the effect of additive of β-lactamases could also result in resistance, despite of the low-level resistance (32 mg/L).

As reported before, CirA was the gateway for cefiderocol entry into bacterial cells (22), hence its deficiency must impede cefiderocol penetration. In this study, we showed the contribution of CirA deficiency on cefiderocol resistance. A base deletion rendered a frameshift mutation, which caused a shortened CirA length (from 657 aa to 443 aa). Actually, this shortened CirA was also observed in cefiderocol-resistant E. cloacae, due to base deletions or insertions (13). Coincidentally, the E. cloacae strain was also NDM-5 positive. Further *in vitro* evolution experiments revealed that NDM-5 facilitates the emergence of CirA mutation, resulting in cefiderocol resistance in Enterobacter cloacae (20). The similar findings were also reported in K. pneumoniae recently (23). Together with our molecular validation in this study, the high-level resistance to cefiderocol was the result of both NDM production and CirA deficiency.

**FIG 4:**
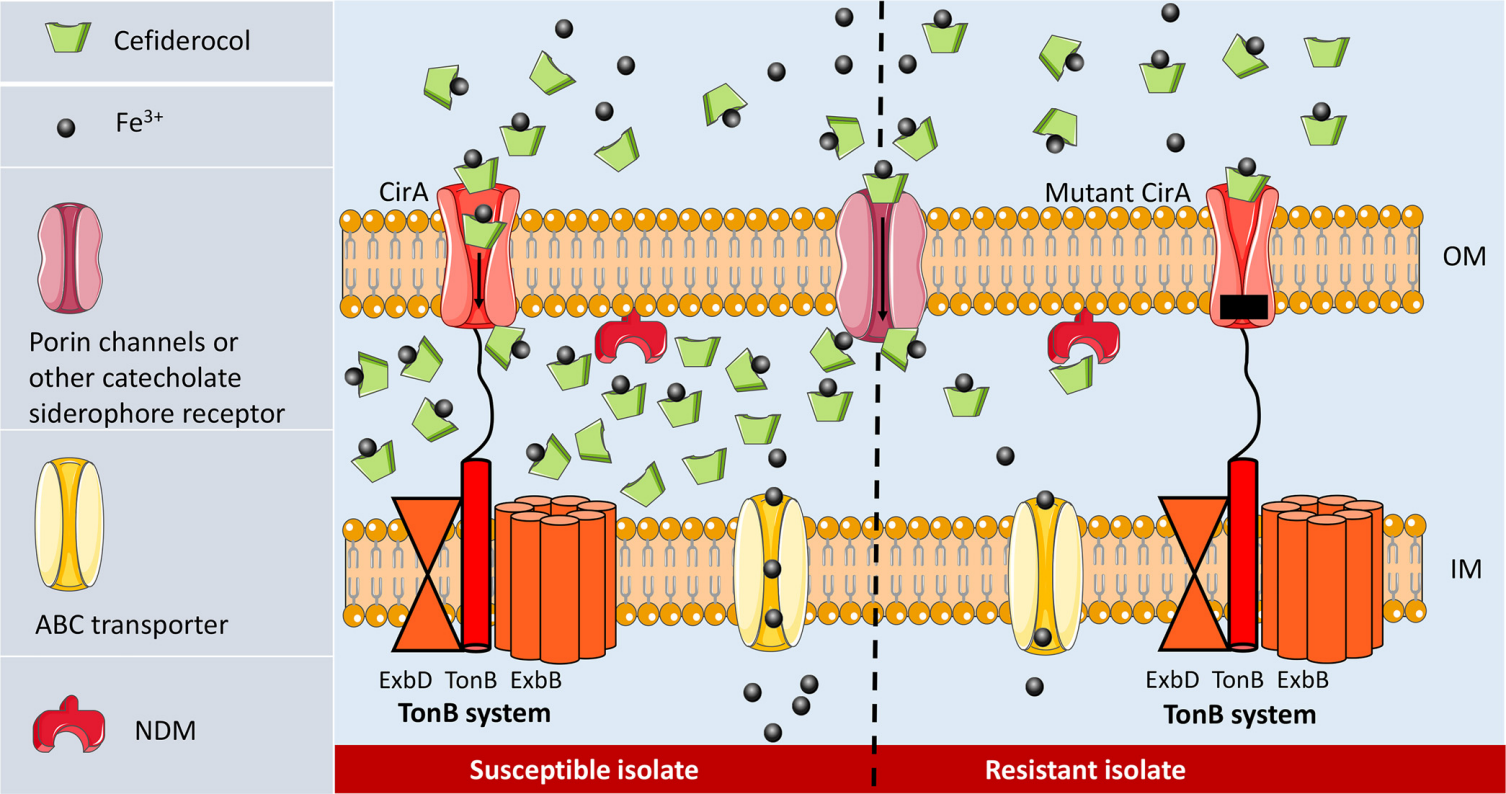
Resistance mechanism schema for high-level cefiderocol resistance. In a NDM-producing CRKP, while CirA functioning normally, plenty of cefiderocol molecules have access to periplasmic space, and thus NDM is insufficient to hydrolyze cefiderocol (left). While the CirA was inactivated (right), the important gateway for cefiderocol entry is switched off. Periplasmic drug concentration then dramatically decreases, making NDM sufficient to hydrolyze cefiderocol.

Actually, CirA is not the unique receptor for cefiderocol entry. The uptake of siderophore (and cefiderocol) into the periplasm requires energy that generated from TonB-ExbB-ExbD system (24). When ferri-siderophores or cefiderocol are bound to outer membrane receptors, TonB system transfer energy to the receptors and induce a conformational change, resulting in internalization of cefiderocol and siderophores (25–27). This active transportation renders a high cefiderocol concentration in periplasm (Fig. 4). In addition to CirA, these TonB-dependent outer membrane transporters (TBDT) include FepA, IroN, FecA, FhuE, IutA, Fiu, Iha, FyuA and FitA in *Enterobacterales*, with FepA, Fiu and CirA as the common catechol receptors. On the other hand, cefiderocol molecules can also cross the outer membrane of by passive diffusion through porin channels (28). Therefore, the transportation of cefiderocol is redundant and not surprisingly when only *cirA* was knocked out in E. coli, cefiderocol MIC was unchanged, whereas the MIC increased 16-fold by the double knockout of *cirA* and *fiu*, though it still remained susceptible (1 mg/L) (14). Consistently, in our test strain D3, mutation of *cirA* had little influence on cefiderocol susceptibility. However, for those NDM-producers (such as AR8416), when CirA is inactivated, NDM is sufficient to hydrolyze decreased cefiderocol in periplasmic space (Fig. 4). These highly resistant strains could not be inhibited even by the combination of cefiderocol and avibactam, a potential therapeutic alternative recently proposed to against carbapenem- and cefiderocol-resistant strains (21).

Clinial analysis of the patients indicate that induced resistance is of concern for cefiderocol. In the phase 3 clinical trial CREDIBLE-CR (29), 12 (15%) patients with a carbapenem-resistant pathogen (including A. baumannii, S. maltophilia, K. pneumoniae, and P. aeruginosa) at baseline treated with cefiderocol had at least a 4-fold increase in cefiderocol MICs. Recently, highly cefiderocol-resistant Enterobacter cloacae was identified after cefiderocol treatment and *cirA* mutations were discovered in resistant isolates (13). It indicated the emergence of cefiderocol resistance after its clinical use. In our study, no cefiderocol or its analogue (ceftazidime and cefepime) was used to treat infections caused by AR8416 and AR8538. Of note, the patients infected by AR8416, which exhibited high-level cefiderocol resistance, experienced extensive exposure to multiple antimicrobial classes, including carbapenems, third-generation cephalosporins, tigecycline, polymyxin B and β-lactamase inhibitors, raising the concerns about the impact of multiple antimicrobial exposure on cefiderocol resistance. It requires further investigation to explore the factors driving the emergence of cefiderocol-resistant strains.

In conclusion, cefiderocol-resistant CRKP strains are emerging from bloodstream infections in patients with hematologic malignancies prior to the approval of cefiderocol clinical use in China. Cefiderocol resistance is mediated by multiple factors, including the deficiency of CirA, metallo- and serine-β-lactamases. High-level cefiderocol resistance could be rendered by the combined effect of NDM expression and CirA deficiency.

## MATERIALS AND METHODS

### Bacterial isolates.

A retrospective multicenter cohort study was performed to evaluate the cefiderocol resistance of CRKP strains isolated from bloodstream infections in patients with hematological malignancies from January 2018 to December 2020. This study covered 15 tertiary referral hospitals from 10 provinces (or cities) in China. K. pneumoniae strains were identified via matrix-assisted laser desorption/ionization mass spectrometry. This study was approved by the ethics committees of Sir Run Run Shaw Hospital (20201231-57).

### Antimicrobial susceptibility testing.

Carbapenem resistance was determined by broth microdilution method with meropenem, imipenem and ertapenem. MICs of cefiderocol were determined using broth microdilution method in iron-depleted cation-adjusted Mueller-Hinton broth (ID-CAMHB) (30). All tests were repeated in triplicates and interpreted following the Clinical and Laboratory Standards Institute 2019 guidelines (cefiderocol MIC: ≤4 mg/L, susceptible; 8 mg/L, intermediate; and ≥16 mg/L, resistant). E. coli ATCC 25922 was served as a quality control strain. To examine the effect of β-lactamase on activity of cefiderocol against these strains, Cefiderocol MICs were determined with or without β-lactamase inhibitors. We used 100 μg/mL of dipicolinic acid (DPA) to inhibit metallo-β-lactamase and 4 μg/mL of AVI to inhibit serine-β-lactamase.

### WGS and sequence analysis.

Genomic DNA was extracted using a Qiagen minikit (Qiagen, Hilden, Germany). We used the TruePrep DNA Library Prep kit v2 (Vazyme Biotech, Nanjing, China) to prepare DNA from AR8416 and AR8538 for next-generation sequencing on the Illumina HiSeq X 10 platform (Illumina, San Diego, CA) with 100-bp paired-end reads and using HiSeq SBS kit v4 (Illumina, San Diego, CA). The quality of the reads was checked using FastQC v.4.5. The two isolates were also sequenced using the MinION platform (Nanopore, Oxford, UK). To assemble whole-genome sequences, the long-read sequencing data from Nanopore sequencing were hybrid assembled with the Illumina sequencing data using Unicycler v.0.4.8. Unicycler uses miniasm to construct a string graph assembly using both the short read contigs and the long reads. Complete and circular chromosome and plasmids were finally obtained. Unicycler uses the SPAdes read error correction module to reduce the number of errors in the short read before SPAdes assembly. Parameter “–only-error-correction” was used in SPAdes assembly. MLST, serotype, antimicrobial resistance and virulence genes were identified using Kleborate (31). Plasmid type was identified by PlasmidFinder-2.0. Synteny plots of plasmid alignment were generated by R v.3.6.2 after pairwise BLASTN comparisons. Complete sequences of the chromosome and plasmids from K. pneumoniae AR8538 and AR8416 have been deposited in GenBank under accession numbers CP081827–CP081831 and CP081815-CP081819, respectively.

### Plasmid curing.

Plasmid curing experiment was performed using sublethal concentration of sodium dodecyl sulfate (SDS) as previously described (32), to confirm whether the *bla*_NDM-5_-bearing plasmid pAR8416-NDM5 has contributed to cefiderocol resistance. Curing of pAR8416-NDM5 was verified by PCR and colonies with only pAR8416-NDM5 cured were collected. All the primers used in this study are shown in Table S1.

### Cloning and sequencing.

The complete sequences of *bla*_NDM-5_, *bla*_NDM-1_, *bla*_KPC-2_, *bla*_IMP-4_, *bla*_OXA-48_ and *bla*_OXA-232_ containing native promoters were identified and amplified by PCR. Purified PCR products were cloned into pCR2.1 vector and transformed into E. coli DH5α. Spread 40 μL of 40 mg/mL X-gal and 40 μL of 100 mM isopropyl β-D-1-thiogalactopyranoside (IPTG) on LB agar plates, which contained 50 mg/L kanamycin. The transformants were then plated on LB agar plates and incubated at 37°C overnight. Pick 10 white or light blue colonies rather than dark blue ones. The recombinant plasmids were verified by PCR using the M13 primers (Table S1) and sequenced on both strands by Sanger sequencing. All operation steps were followed by the instruction of TOPO TA Cloning kit.

### Site-specific mutagenesis for *cirA*.

To explore the impact of termination of *cirA* coding, we performed single-base deletion mutation as present in AR8416 for *cirA* via a CRISPR-Cas9-mediated genome-editing method (33). Generally, the single guide RNA (sgRNA) directs the Cas9 protein to a target sequence in the presence of a 5′-NGG-3′ protospacer adjacent motif (PAM). Cas9 nuclease then cleaves the target sequence to cause a double-strand break. To repair the break, homologous recombination occurs with the presence of exogenously supplied donor DNA repair templates.

Specifically, a 20 bp-spacer sequence before a PAM site (5′-NGG-3′) in the target sequence was ligated into plasmid pSGKP, which expressed the sgRNA. Two reversed *BsaI* sites were inserted between the promoter and the sgRNA scaffold for assembly of spacers (33). Cotransform the spacer introduced plasmid pSGKP and the donor DNA (containing deletion mutation) into the L-arabinose induced recipient cells harboring the pCasKP plasmid, which expressed the Cas9 protein and lambda Red recombination system. The MH agar plate containing 50 μg/mL apramycin and 100 μg/mL hygromycin was used to screen the positive transformants at 30°C overnight. The successful mutant was verified by PCR and sanger sequencing. Of note, a silent mutation should be introduced in the PAM site on donor DNA to prevent the cutting of Cas9 when performing complementation. Primers used in this study are shown in Table S1. Plasmid pCasKP and pSGKP contained the temperature-sensitive replicon *repA101*(Ts) and the sucrose-sensitive gene *sacB*, respectively (33). To cure these two plasmids after successful editing, bacterial cells were streaked onto a MH agar plate containing 5% sucrose and incubated at 37°C overnight. Survival colonies were plated onto MH plates with or without the supplementation of apramycin or hygromycin, respectively. The colonies with the successful curing of both plasmids could only grow on the plate without antibiotic.

### Data availability.

Complete sequences of the chromosome and plasmids from K. pneumoniae AR8538 and AR8416 have been deposited in GenBank under accession numbers CP081827–CP081831 and CP081815-CP081819, respectively.

## References

[1] Trecarichi EM, Pagano L, Martino B, Candoni A, Di Blasi R, Nadali G, Fianchi L, Delia M, Sica S, Perriello V, Busca A, Aversa F, Fanci R, Melillo L, Lessi F, Del Principe MI, Cattaneo C, Tumbarello M, Haematologic Malignancies Associated Bloodstream Infections Surveillance (HEMABIS) registry - Sorveglianza Epidemiologica Infezioni Funginein Emopatie Maligne (SEIFEM) group, Italy. 2016. Bloodstream infections caused by Klebsiella pneumoniae in onco-hematological patients: clinical impact of carbapenem resistance in a multicentre prospective survey. Am J Hematol 91:1076–1081. doi:10.1002/ajh.24489. 27428072

[2] Mert D, Ceken S, Iskender G, Iskender D, Merdin A, Duygu F, Ertek M, Altuntas F. 2019. Epidemiology and mortality in bacterial bloodstream infections in patients with hematologic malignancies. J Infect Dev Ctries 13:727–735. doi:10.3855/jidc.11457. 32069257

[3] Rodríguez-Baño J, Gutiérrez-Gutiérrez B, Machuca I, Pascual A. 2018. Treatment of infections caused by extended-spectrum-beta-lactamase-, AmpC-, and carbapenemase-producing Enterobacteriaceae. Clin Microbiol Rev 31. doi:10.1128/CMR.00079-17. PMC5967687 29444952

[4] van Duin D, Doi Y. 2017. The global epidemiology of carbapenemase-producing Enterobacteriaceae. Virulence 8:460–469. doi:10.1080/21505594.2016.1222343. 27593176 PMC5477705

[5] Laxminarayan R, Van Boeckel T, Frost I, Kariuki S, Khan EA, Limmathurotsakul D, Larsson DGJ, Levy-Hara G, Mendelson M, Outterson K, Peacock SJ, Zhu YG. 2020. The Lancet Infectious Diseases Commission on antimicrobial resistance: 6 years later. Lancet Infect Dis 20:e51–e60. doi:10.1016/S1473-3099(20)30003-7. 32059790

[6] Shirley M. 2018. Ceftazidime-avibactam: a review in the treatment of serious Gram-Negative Bacterial Infections. Drugs 78:675–692. doi:10.1007/s40265-018-0902-x. 29671219

[7] Zhanel GG, Golden AR, Zelenitsky S, Wiebe K, Lawrence CK, Adam HJ, Idowu T, Domalaon R, Schweizer F, Zhanel MA, Lagacé-Wiens PRS, Walkty AJ, Noreddin A, Lynch Iii JP, Karlowsky JA. 2019. Cefiderocol: a siderophore cephalosporin with activity against carbapenem-resistant and multidrug-resistant Gram-negative bacilli. Drugs 79:271–289. doi:10.1007/s40265-019-1055-2. 30712199

[8] Yamano Y. 2019. In vitro activity of cefiderocol against a broad range of clinically important Gram-negative bacteria. Clin Infect Dis 69:S544–s551. doi:10.1093/cid/ciz827. 31724049 PMC6853761

[9] Abdul-Mutakabbir JC, Alosaimy S, Morrisette T, Kebriaei R, Rybak MJ. 2020. Cefiderocol: a novel siderophore cephalosporin against multidrug-resistant gram-negative pathogens. Pharmacotherapy 40:1228–1247. doi:10.1002/phar.2476. 33068441

[10] Kawai A, McElheny CL, Iovleva A, Kline EG, Sluis-Cremer N, Shields RK, Doi Y. 2020. Structural basis of reduced susceptibility to ceftazidime-avibactam and cefiderocol in enterobacter cloacae due to AmpC R2 loop deletion. Antimicrob Agents Chemother 64. doi:10.1128/AAC.00198-20. PMC7318025 32284381

[11] Shields RK, Iovleva A, Kline EG, Kawai A, McElheny CL, Doi Y. 2020. Clinical evolution of AmpC-mediated ceftazidime-avibactam and cefiderocol resistance in Enterobacter cloacae complex following exposure to cefepime. Clin Infect Dis 71:2713–2716. doi:10.1093/cid/ciaa355. 32236408 PMC7744991

[12] Kohira N, Hackel MA, Ishioka Y, Kuroiwa M, Sahm DF, Sato T, Maki H, Yamano Y. 2020. Reduced susceptibility mechanism to cefiderocol, a siderophore cephalosporin, among clinical isolates from a global surveillance programme (SIDERO-WT-2014). J Glob Antimicrob Resist 22:738–741. doi:10.1016/j.jgar.2020.07.009. 32702396

[13] Klein S, Boutin S, Kocer K, Fiedler MO, Störzinger D, Weigand MA, Tan B, Richter D, Rupp C, Mieth M, Mehrabi A, Hackert T, Zimmermann S, Heeg K, Nurjadi D. 2021. Rapid development of cefiderocol resistance in carbapenem-resistant Enterobacter cloacae during therapy is associated with heterogeneous mutations in the catecholate siderophore receptor cira. Clin Infect Dis. doi:10.1093/cid/ciab511. PMC8906715 34079986

[14] Ito A, Sato T, Ota M, Takemura M, Nishikawa T, Toba S, Kohira N, Miyagawa S, Ishibashi N, Matsumoto S, Nakamura R, Tsuji M, Yamano Y. 2018. In vitro antibacterial properties of cefiderocol, a novel siderophore cephalosporin, against Gram-negative bacteria. Antimicrob Agents Chemother 62. doi:10.1128/AAC.01454-17. PMC5740388 29061741

[15] Buchanan SK, Lukacik P, Grizot S, Ghirlando R, Ali MM, Barnard TJ, Jakes KS, Kienker PK, Esser L. 2007. Structure of colicin I receptor bound to the R-domain of colicin Ia: implications for protein import. EMBO J 26:2594–2604. doi:10.1038/sj.emboj.7601693. 17464289 PMC1868905

[16] Wu W, Feng Y, Tang G, Qiao F, McNally A, Zong Z. 2019. NDM metallo-β-lactamases and their bacterial producers in health care settings. Clin Microbiol Rev 32. doi:10.1128/CMR.00115-18. PMC6431124 30700432

[17] Syed YY. 2021. Cefiderocol: a review in serious Gram-negative bacterial infections. Drugs 81:1559–1571. doi:10.1007/s40265-021-01580-4. 34427896 PMC8383240

[18] Hackel MA, Tsuji M, Yamano Y, Echols R, Karlowsky JA, Sahm DF. 2018. In vitro activity of the siderophore cephalosporin, cefiderocol, against carbapenem-nonsusceptible and multidrug-resistant isolates of Gram-negative bacilli collected worldwide in 2014 to 2016. Antimicrob Agents Chemother 62. doi:10.1128/AAC.01968-17. PMC5786755 29158270

[19] Kazmierczak KM, Tsuji M, Wise MG, Hackel M, Yamano Y, Echols R, Sahm DF. 2019. In vitro activity of cefiderocol, a siderophore cephalosporin, against a recent collection of clinically relevant carbapenem-non-susceptible Gram-negative bacilli, including serine carbapenemase- and metallo-β-lactamase-producing isolates (SIDERO-WT-2014 Study). Int J Antimicrob Agents 53:177–184. doi:10.1016/j.ijantimicag.2018.10.007. 30395986

[20] Nurjadi D, Kocer K, Chanthalangsy Q, Klein S, Heeg K, Boutin S. 2022. New Delhi metallo-beta-lactamase facilitates the emergence of cefiderocol resistance in Enterobacter cloacae. Antimicrob Agents Chemother 66. doi:10.1128/aac.02011-21. PMC8846454 34871093

[21] Poirel L, Sadek M, Nordmann P. 2021. Contribution of PER-type and NDM-type ß-lactamases to cefiderocol resistance in Acinetobacter baumannii. Antimicrob Agents Chemother 65. doi:10.1128/AAC.00877-21. PMC8448131 34252309

[22] Tillotson GS. 2016. Trojan horse antibiotics: a novel way to circumvent Gram-negative bacterial resistance? Infect Dis (Auckl) 9:45–52. doi:10.4137/IDRT.S31567. 27773991 PMC5063921

[23] McElheny CL, Fowler EL, Iovleva A, Shields RK, Doi Y. 2021. In Vitro Evolution of Cefiderocol Resistance in an NDM-Producing Klebsiella pneumoniae Due to Functional Loss of CirA. Microbiol Spectr 9:e0177921. doi:10.1128/Spectrum.01779-21. 34756080 PMC8579844

[24] Higgs PI, Larsen RA, Postle K. 2002. Quantification of known components of the Escherichia coli TonB energy transduction system: TonB, ExbB, ExbD and FepA. Mol Microbiol 44:271–281. doi:10.1046/j.1365-2958.2002.02880.x. 11967085

[25] Moeck GS, Coulton JW. 1998. TonB-dependent iron acquisition: mechanisms of siderophore-mediated active transport. Mol Microbiol 28:675–681. doi:10.1046/j.1365-2958.1998.00817.x. 9643536

[26] Postle K, Larsen RA. 2007. TonB-dependent energy transduction between outer and cytoplasmic membranes. Biometals 20:453–465. doi:10.1007/s10534-006-9071-6. 17225934

[27] Krewulak KD, Vogel HJ. 2011. TonB or not TonB: is that the question? Biochem Cell Biol 89:87–97. doi:10.1139/o10-141. 21455261

[28] El-Lababidi RM, Rizk JG. 2020. Cefiderocol: a siderophore cephalosporin. Ann Pharmacother 54:1215–1231. doi:10.1177/1060028020929988. 32522005

[29] Bassetti M, Echols R, Matsunaga Y, Ariyasu M, Doi Y, Ferrer R, Lodise TP, Naas T, Niki Y, Paterson DL, Portsmouth S, Torre-Cisneros J, Toyoizumi K, Wunderink RG, Nagata TD. 2021. Efficacy and safety of cefiderocol or best available therapy for the treatment of serious infections caused by carbapenem-resistant Gram-negative bacteria (CREDIBLE-CR): a randomised, open-label, multicentre, pathogen-focused, descriptive, phase 3 trial. Lancet Infect Dis 21:226–240. doi:10.1016/S1473-3099(20)30796-9. 33058795

[30] Clinical and Laboratory Standards Institute. 2020. Performance standards for antimicrobial susceptibility testing. CLSI supplement M100. 30th ed Wayne, PA, USA.

[31] Lam MMC, Wick RR, Watts SC, Cerdeira LT, Wyres KL, Holt KE. 2021. A genomic surveillance framework and genotyping tool for Klebsiella pneumoniae and its related species complex. Nat Commun 12:4188. doi:10.1038/s41467-021-24448-3. 34234121 PMC8263825

[32] El-Mansi M, Anderson KJ, Inche CA, Knowles LK, Platt DJ. 2000. Isolation and curing of the Klebsiella pneumoniae large indigenous plasmid using sodium dodecyl sulphate. Res Microbiol 151:201–208. doi:10.1016/s0923-2508(00)00140-6. 10865947

[33] Wang Y, Wang S, Chen W, Song L, Zhang Y, Shen Z, Yu F, Li M, Ji Q. 2018. CRISPR-Cas9 and CRISPR-assisted cytidine deaminase enable precise and efficient genome editing in Klebsiella pneumoniae. Appl Environ Microbiol 84. doi:10.1128/AEM.01834-18. PMC6238054 30217854

