# Supplementary material for: Emergence of High-Level Cefiderocol Resistance in Carbapenem-Resistant Klebsiella pneumoniae from Bloodstream Infections in Patients with Hematologic Malignancies in China
Source: Microbiol Spectr. 2022 Mar 24;10(2):e00084-22. doi: 10.1128/spectrum.00084-22 (PMC9045219; doi:10.1128/spectrum.00084-22)
Supplement: SUPPLEMENTAL FILE 1 — Supplemental material. Download SPECTRUM00084-22_Supp_1_seq8.pdf, PDF file, 0.7 MB [file spectrum00084-22_supp_1_seq8.pdf]

Supplemental Online Content

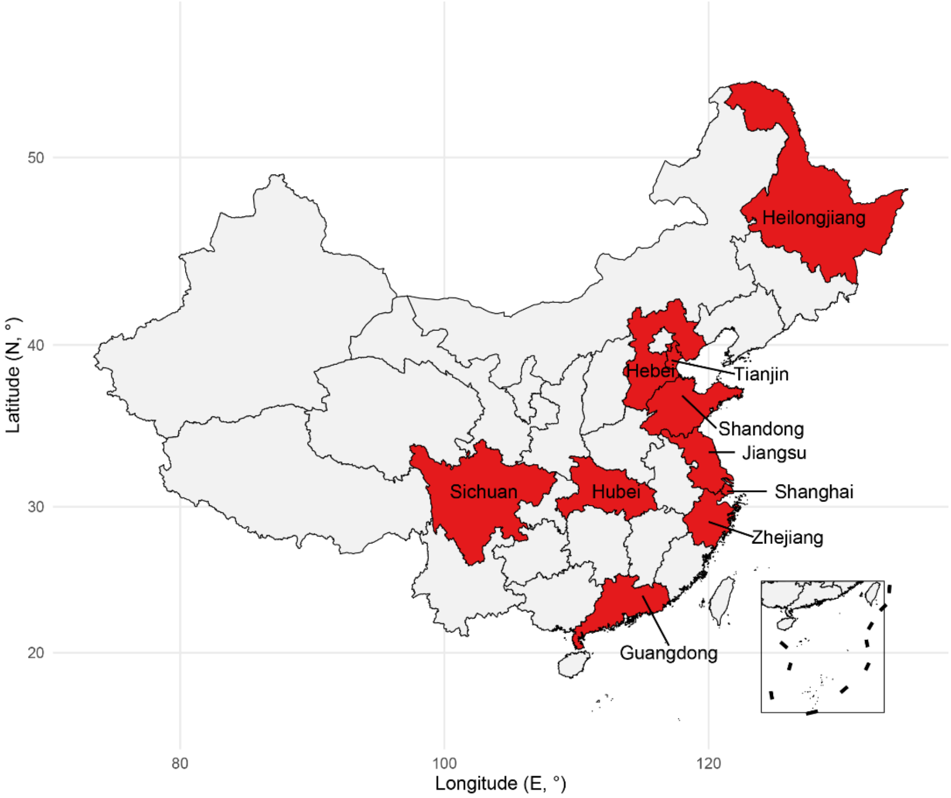

Figure S1 *K. pneumoniae* strains in this study were identified from 10 provinces/cities, which are highlighted with red.

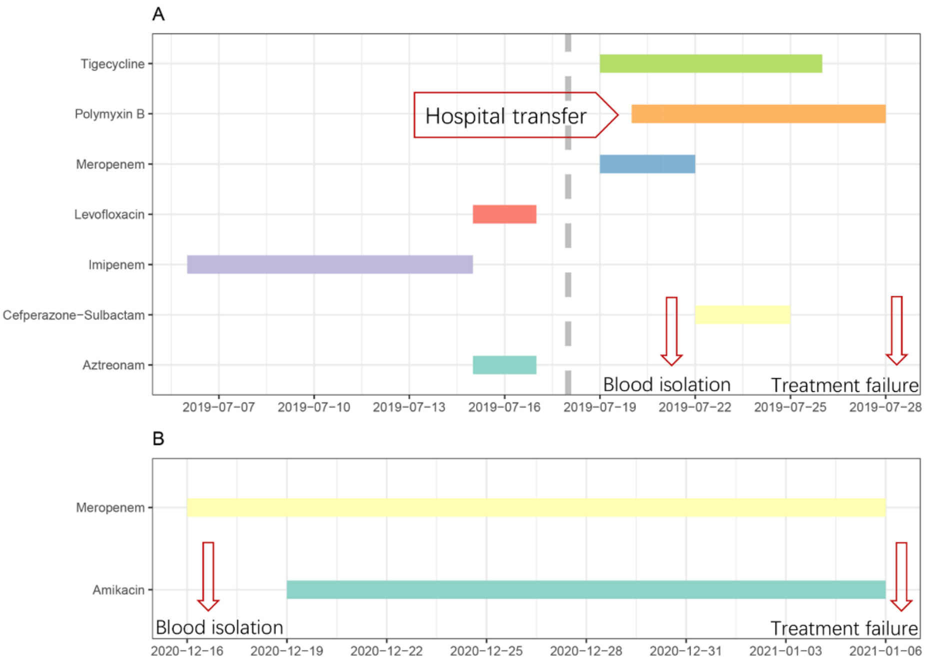

Figure S2 Antimicrobials treatment during hospitalization for patients infected by *K. pneumoniae* AR8416 and AR8538.

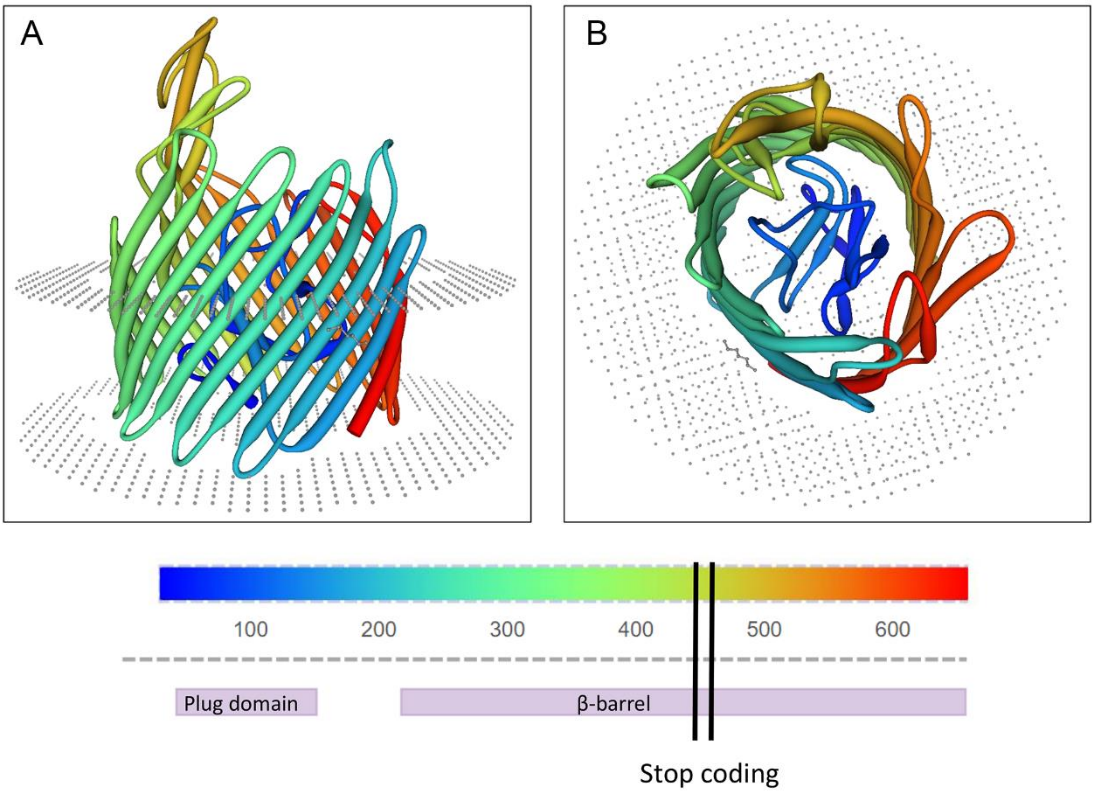

Figure S3 Structure of CirA and its deficiency. (A) side view (B) top (extracellular) view. The plug domain is indicated with blue and the rest was β-barrel. An early stop codon occurs at position 444 of CirA. The CirA structure was modeled by SWISS-MODEL (https://swissmodel.expasy.org/).

Table S1 Primers used in this study

Primer name	Sequences (5'-3')	Usage
NDM5-full-F	TGGCTTTTGAAACTGTCGCA	Cloning
NDM5-full-R	GATGGCAGATTGGGGGTGAC	Cloning
NDM1-full-F	CATGGCATCGAGATCATCCA	Cloning
NDM1-full-R	ACCTCATGTTTGAATTCGCC	Cloning
KPC2-full-F	CAGTCGTCATAACACGCCGC	Cloning
KPC2-full-R	GCGGCCTGATTACATCCGGC	Cloning
IMP-4-full-F	CCGAGGAAAGATCCAATGGC	Cloning
IMP-4-full-R	CAGCAACGATGTTACGCAGC	Cloning
OXA-48-full-F	ATATCAATGATTTTCTGGTGC	Cloning
OXA-48-full-R	CTCAGGCTAAAAGTTAACGC	Cloning
OXA-232-full-F	AAATAATGTTACAATGTGTG	Cloning
OXA-232-full-R	CCATATATCTTTACAGCTTA	Cloning
cirA1-full-F	TCGTAGACAGGGTAAGGCGA	Mutation confirmation
cirA1-full-R	TATCGATCAGGGCGACAACG	Mutation confirmation

spacer-cirA-stop-F	TAGTCGACGATGCTGCACGAGCCG	Spacer for cirA mutation
spacer-cirA-stop-R	AAACCGGCTCGTGCAGCATCGTCG	Spacer for cirA mutation
donor-cirA-stop-F	GCTGGTTTCCGGTTTCAGATCCGGGTTACC GACGATGCTGCACGAGCGCGACAGGAGTT	Donar for cirA mutation
donor-cirA-stop-R	GGCGGCCAGTCAACGTC	Donar for cirA mutation
pCasKP-conf-F	GGGAAATACAGACCGCCACA	pCasKP confirmation
pCasKP-conf-R	TGAAGCTGATAGGGGAGCCT	pCasKP confirmation
pSGKP-conf-F	AGGATTTGCAGACTACGGGC	pSGKP confirmation
pSGKP-conf-R	TGTGTGGAATTGTGAGCGGA	pSGKP confirmation
AR8416-Plasmid2-F	CCGGCGAAGTTGTTAAGCTG	Curing confirmation
AR8416-Plasmid2-R	TTCATCGCTGAGAGGGCTTC	Curing confirmation
AR8416-plasmid3-F	AATTTCTCCAAAGCCCGCGA	Curing confirmation
AR8416-plasmid3-R	GTCGGCATCGGCCTGATTAT	Curing confirmation
AR8416-plasmid4-F	GGACGACCATCGAGTTCTGG	Curing confirmation
AR8416-plasmid4-R	CACTACCTGAGTTCCGCGAC	Curing confirmation
M13-F	GTAAAACGACGGCCAG	Cloning confirmation
M13-R	CAGGAAACAGCTATGAC	Cloning confirmation
BsaI sites were indicated with underlines for spacer sequences.		

Tables S2 Clinical data and cefiderocol susceptibility of the 86 CRKP strains

Isolates Name	Species	Cefiderocol MIC (mg/L)	Diagnosis	City
AR8214	*Klebsiella pneumoniae*	0.5	Acute T lymphoblastic leukemia	Harbin
AR8215	*Klebsiella quasipneumoniae*	2	Acute myelogenous leukemia（AML）	Harbin
AR8217	*Klebsiella pneumoniae*	0.25	Acute lymphoblastic leukemia	Harbin
AR8221	*Klebsiella pneumoniae*	0.5	Lymphoma	Harbin
AR8229	*Klebsiella pneumoniae*	2	Acute myelogenous leukemia	Wenzhou
AR8230	*Klebsiella pneumoniae*	0.06	Malignant B cell lymphoma	Wenzhou
AR8231	*Klebsiella pneumoniae*	0.25	Acute lymphoblastic leukemia	Wenzhou
AR8232	*Klebsiella pneumoniae*	0.06	Acute monocytic leukemia	Wenzhou
AR8234	*Klebsiella quasipneumoniae*	0.06	Acute lymphoblastic leukemia	Wenzhou

AR8235	*Klebsiella pneumoniae*	0.25	Acute myelogenous leukemia	Wenzhou
AR8236	*Klebsiella pneumoniae*	0.25	Acute myelogenous leukemia	Wenzhou
AR8237	*Klebsiella pneumoniae*	0.25	Diffuse large B cell lymphoma	Wenzhou
AR8287	*Klebsiella quasipneumoniae*	2	Acute leukemia	Jinan
AR8288	*Klebsiella pneumoniae*	2	Acute myelogenous leukemia	Jinan
AR8302	*Klebsiella pneumoniae*	2	Chronic myelogenous leukemia	Hangzhou
AR8303	*Klebsiella pneumoniae*	0.5	Acute lymphoblastic leukemia	Hangzhou
AR8304	*Klebsiella pneumoniae*	1	Acute monocytic leukemia	Hangzhou
AR8305	*Klebsiella pneumoniae*	0.25	Acute T lymphoblastic leukemia	Hangzhou
AR8308	*Klebsiella pneumoniae*	4	Acute monocytic leukemia	Hangzhou
AR8309	*Klebsiella variicola*	1	AML with maturation	Hangzhou
AR8310	*Klebsiella pneumoniae*	4	Non-Hodgkin lymphoma	Hangzhou
AR8313	*Klebsiella pneumoniae*	1	Acute mixed-lineage leukemia	Hangzhou
AR8314	*Klebsiella pneumoniae*	1	Acute lymphoblastic leukemia	Hangzhou
AR8315	*Klebsiella pneumoniae*	1	Acute lymphoblastic leukemia	Hangzhou
AR8319	*Klebsiella pneumoniae*	4	Chronic myelogenous leukemia	Hangzhou
AR8324	*Klebsiella pneumoniae*	2	Acute myelogenous leukemia	Hangzhou
AR8325	*Klebsiella pneumoniae*	4	Acute B lymphoblastic leukemia	Hangzhou
AR8331	*Klebsiella pneumoniae*	4	Diffuse large B cell lymphoma	Hangzhou
AR8334	*Klebsiella pneumoniae*	8	Acute myelogenous leukemia	Hangzhou
AR8335	*Klebsiella pneumoniae*	8	Acute myelogenous leukemia	Hangzhou
AR8336	*Klebsiella pneumoniae*	4	Acute myelogenous leukemia	Hangzhou
AR8345	*Klebsiella pneumoniae*	0.5	Acute lymphoblastic leukemia	Hangzhou
AR8381	*Klebsiella pneumoniae*	1	AML with maturation	Hangzhou
AR8388	*Klebsiella pneumoniae*	2	Follicular lymphoma	Hangzhou
AR8393	*Klebsiella pneumoniae*	0.125	Acute monocytic leukemia	Shanghai
AR8394	*Klebsiella pneumoniae*	0.25	Follicular lymphoma	Shanghai
AR8396	*Klebsiella pneumoniae*	1	Acute lymphoblastic leukemia	Shanghai
AR8398	*Klebsiella pneumoniae*	0.5	Multiple myeloma	Shanghai
AR8399	*Klebsiella pneumoniae*	0.25	Acute B lymphoblastic leukemia	Shanghai
AR8403	*Klebsiella pneumoniae*	2	Acute myelogenous leukemia	Shanghai
AR8406	*Klebsiella pneumoniae*	0.5	Acute lymphoblastic leukemia	Shanghai
AR8407	*Klebsiella pneumoniae*	0.25	Diffuse large B cell lymphoma	Shanghai
AR8408	*Klebsiella pneumoniae*	0.5	Diffuse large B cell lymphoma	Shanghai
AR8411	*Klebsiella pneumoniae*	0.25	Acute lymphoblastic leukemia	Shanghai
AR8412	*Klebsiella pneumoniae*	1	Burkitt lymphoma	Shanghai
AR8413	*Klebsiella pneumoniae*	0.5	Burkitt lymphoma	Shanghai
AR8414	*Klebsiella variicola*	0.25	NK cell lymphoma	Shanghai
AR8416	*Klebsiella pneumoniae*	>256	Acute lymphoblastic leukemia	Shanghai
AR8419	*Klebsiella pneumoniae*	0.06	Diffuse large B cell lymphoma	Shanghai
AR8423	*Klebsiella pneumoniae*	2	Diffuse large B cell lymphoma	Hangzhou
AR8424	*Klebsiella pneumoniae*	2	Diffuse large B cell lymphoma	Hangzhou

AR8447	*Klebsiella pneumoniae*	0.5	Acute mixed-lineage leukemia	Guangzhou
AR8452	*Klebsiella pneumoniae*	0.25	Multiple myeloma	Chengdu
AR8457	*Klebsiella pneumoniae*	0.125	Acute lymphoblastic leukemia	Chengdu
AR8458	*Klebsiella pneumoniae*	0.25	AML with maturation	Chengdu
AR8459	*Klebsiella pneumoniae*	0.25	Lymphoma	Chengdu
AR8471	*Klebsiella pneumoniae*	0.125	Acute monocytic leukemia	Chengdu
AR8476	*Klebsiella pneumoniae*	0.125	Acute T lymphoblastic leukemia	Shijiazhuang
AR8479	*Klebsiella pneumoniae*	0.5	Acute promyelocytic leukemia	Shijiazhuang
AR8480	*Klebsiella pneumoniae*	0.125	Acute lymphoblastic leukemia	Shijiazhuang
AR8481	*Klebsiella pneumoniae*	0.5	Acute myelogenous leukemia	Shijiazhuang
AR8484	*Klebsiella pneumoniae*	0.5	NK/T cell lymphoma	Shanghai
AR8487	*Klebsiella pneumoniae*	0.25	Acute myelogenous leukemia	Hangzhou
AR8488	*Klebsiella pneumoniae*	0.25	Acute lymphoblastic leukemia	Hangzhou
AR8489	*Klebsiella pneumoniae*	0.125	Acute myelogenous leukemia	Hangzhou
AR8490	*Klebsiella pneumoniae*	0.25	Non-Hodgkin lymphoma	Hangzhou
AR8498	*Klebsiella pneumoniae*	0.25	Acute T lymphoblastic leukemia	Shenyang
AR8499	*Klebsiella pneumoniae*	0.25	Chronic myelomonocytic leukemia	Shenyang
AR8500	*Klebsiella pneumoniae*	0.5	Acute lymphoblastic leukemia	Shenyang
AR8507	*Klebsiella pneumoniae*	2	AML with maturation	Hangzhou
AR8508	*Klebsiella pneumoniae*	0.5	Acute lymphoblastic leukemia	Hangzhou
AR8509	*Klebsiella pneumoniae*	4	AML with maturation	Hangzhou
AR8510	*Klebsiella pneumoniae*	2	Acute myelogenous leukemia	Hangzhou
AR8513	*Klebsiella pneumoniae*	1	Acute lymphoblastic leukemia	Hangzhou
AR8514	*Klebsiella pneumoniae*	1	Acute mixed-lineage leukemia	Hangzhou
AR8518	*Klebsiella pneumoniae*	2	Acute lymphoblastic leukemia	Hangzhou
AR8523	*Klebsiella pneumoniae*	1	Acute myelogenous leukemia	Hangzhou
AR8529	*Klebsiella pneumoniae*	2	Multiple myeloma	Suzhou
AR8530	*Klebsiella pneumoniae*	2	Angioimmunoblastic T cell lymphoma	Suzhou
AR8531	*Klebsiella pneumoniae*	2	Acute lymphoblastic leukemia	Suzhou
AR8532	*Klebsiella pneumoniae*	1	Acute leukemia	Suzhou
AR8533	*Klebsiella pneumoniae*	2	Acute myelogenous leukemia	Suzhou
AR8534	*Klebsiella pneumoniae*	0.5	Acute leukemia	Suzhou
AR8535	*Klebsiella pneumoniae*	1	Acute lymphoblastic leukemia	Suzhou
AR8537	*Klebsiella pneumoniae*	0.5	Acute B lymphoblastic leukemia	Suzhou
AR8538	*Klebsiella quasipneumoniae*	32	Acute lymphoblastic leukemia	Suzhou
All the strains were identified as *K. pneumoniae* via matrix-assisted laser desorption/ionization mass spectrometry and then verified by Kleborate with genome data. All the strains belong to *K. pneumoniae* species complex [1].				

Reference

1. Wyres KL, Lam MMC, Holt KE. Population genomics of Klebsiella pneumoniae. *Nat Rev Microbiol* 2020.
